# Testing an attachment- and trauma-informed intervention approach for parents and young children after interparental violence: protocol for a randomized controlled trial

**DOI:** 10.1186/s13063-022-06902-9

**Published:** 2022-12-05

**Authors:** Sabine van der Asdonk, Ashwina R. Kesarlal, Carlo Schuengel, Nina Draaisma, Carlijn de Roos, Karine Zuidgeest, Ralph C. A. Rippe, Lenneke R. A. Alink

**Affiliations:** 1 Institute of Education and Child Studies, Faculty of Social and Behavioural Sciences, Leiden University, Leiden, the Netherlands; 2 Clinical Child & Family Studies, Faculty of Behavioral and Movement Sciences, Vrije Universiteit, Amsterdam, the Netherlands; 3 NIKA Nederland, Haarlem, the Netherlands; 4 Department of Child and Adolescent Psychiatry, Amsterdam University Medical Centre, Amsterdam, the Netherlands

**Keywords:** Domestic violence, Interparental violence, Trauma, PTSD, Attachment, RCT, EMDR therapy, Intervention, Early childhood, Video feedback

## Abstract

**Background:**

Interparental violence has persistent adverse effects on victimized parents and children. Young children, including infants and toddlers, are at particular risk to develop long-lasting negative outcomes, and yet specific evidence on effective intervention approaches for this vulnerable group is still lacking. This study will test the effectiveness of an attachment- and trauma-informed intervention approach in a sample of parent-child dyads who have experienced severe interparental violence. We test the individual and combined effects of two interventions: (1) “Nederlandse Interventie Kortdurend op Atypisch oudergedrag” (NIKA; Dutch, short-term intervention focused on atypical parenting behavior) aimed at improving the attachment relationship and (2) eye movement desensitization and reprocessing (EMDR) therapy aimed at reducing parental post-traumatic stress disorder (PTSD) symptoms.

**Methods:**

This study uses a multicenter randomized controlled design across multiple domestic violence shelters in the Netherlands. We aim to recruit 150 parent-child dyads with children aged between 0.5 and 6 years old. The study design consists of two phases. During the first phase for testing the effect of NIKA only, eligible dyads are randomly allocated to either NIKA or a waitlist usual care group. A pre-test is conducted prior to the treatment period and a post-test takes place directly afterwards (6 weeks after the pre-test). Phase 2 follows directly for testing the effects of EMDR and the combination of NIKA and EMDR. Parents who report clinical PTSD symptoms are randomly allocated to either EMDR therapy or a waitlist usual care group. Parents who do not report clinical PTSD symptoms only receive care as usual. Six weeks later, a post-test of phase 2 is conducted for all participating dyads. Primary study outcomes are disrupted parenting behavior, sensitive parenting behavior, and parental PTSD symptoms. Secondary study outcomes include PTSD symptoms and behavioral and emotional problems of the child.

**Discussion:**

This study will inform and enhance the clinical field by providing new insights regarding effective treatment combinations for traumatized parents and their young children after interparental violence.

**Trial registration:**

Netherlands Trial Register (NTR) NL9179. Registered 7 January 2021

## Administrative information

Note: the numbers in curly brackets in this protocol refer to SPIRIT checklist item numbers. The order of the items has been modified to group similar items (see http://www.equator-network.org/reporting-guidelines/spirit-2013-statement-defining-standard-protocol-items-for-clinical-trials/).

**Table:** 

Title {1}	Testing an attachment- and trauma-informed intervention approach for parents and young children after interparental violence: protocol for a randomized controlled trial
Trial registration {2a and 2b}.	Netherlands Trial Register (NTR): NL9179
Protocol version {3}	Issue Date: 23/08/2022 Protocol number: 01
Funding {4}	This study is funded by ZonMw (The Netherlands Organisation for Health Research and Development), project number 1026001 1910001. The Institute of Education and Child Studies from Leiden University provides additional financial support.
Author details {5a}	SvdA, ARK, ND, RCAR, and LRAA are affiliated with the Institute of Education and Child Studies, Faculty of Social and Behavioural Sciences, Leiden University, the Netherlands CS is affiliated with Clinical Child & Family Studies, Faculty of Behavioral and Movement Sciences, Vrije Universiteit, Amsterdam, the Netherlands ND and KZ are affiliated with NIKA Nederland, Haarlem, the Netherlands CdR is affiliated with Amsterdam University Medical Centre, Department of Child and Adolescent Psychiatry, Amsterdam, the Netherlands
Name and contact information for the trial sponsor {5b}	Trial sponsor: Institute of Education and Child Studies, Faculty of Social and Behavioural Sciences, Leiden University, Wassenaarseweg 52, 2333 AK Leiden, the Netherlands.
Role of sponsor {5c}	The sponsor is responsible for the study design, data collection, data management, data analyses, interpretation, and results dissemination. The funder monitors the project through yearly reports and evaluations, but had no role in the design of this study and will have no role in the data collection, analyses, interpretation of the data, or decision to submit results.

## Introduction

### Background and rationale {6a}

Domestic violence, including child maltreatment, is often traumatizing for its victims and can have detrimental effects on all levels of development, ranging from epigenesis and stress dysregulation to decreased cognitive capacities, increased psychopathology, and somatic health problems [1–3]. Furthermore, meta-analytical research shows that victimized children are at increased risk to become perpetrators of maltreatment themselves later in life [4, 5]. Domestic violence also comes with high economic costs for society [6–8]. Effective intervention programs to prevent and stop domestic violence and its consequences are thus of utmost importance. One of the most vulnerable groups consists of families with traumatized parents and children, who are both victims of domestic violence. This is a considerable group; almost half of the children who are victims of maltreatment live in families characterized by other types of domestic violence, most of which takes place between parents or between a parent and an ex-partner [9].

Intervention studies that specifically target families after interparental violence are scarce, and existing studies are heterogeneous in terms of the focus of the intervention program, the sample that is included, and the methodological quality of the study [10, 11]. This makes it hard to derive strong conclusions on which intervention approach is most beneficial for this population. In addition, even fewer studies have specifically targeted young children including infants and toddlers, despite the vulnerability of this group of children for long-lasting consequences of traumatic experiences (e.g., [12, 13]). A promising but yet untested idea in this population is to offer integral treatment of psychopathology related to parental and child traumatization and disruption in the parent-child relationship. The aim of the current study is to gain insight in the effectiveness of combining two interventions in a population of victimized parents and their young children (aged 0.5–6 years) after interparental violence: (1) “Nederlandse Interventie Kortdurend op Atypisch oudergedrag” (NIKA; Dutch, short-term intervention focused on atypical parenting behavior), a video-feedback intervention aimed at enhancing the parent-child relationship [14], and (2) eye movement desensitization and reprocessing (EMDR) therapy aimed at reduction of PTSD symptoms in parents.

#### Treatment focus on enhancing the parent-child relationship

**Fig. 1:**
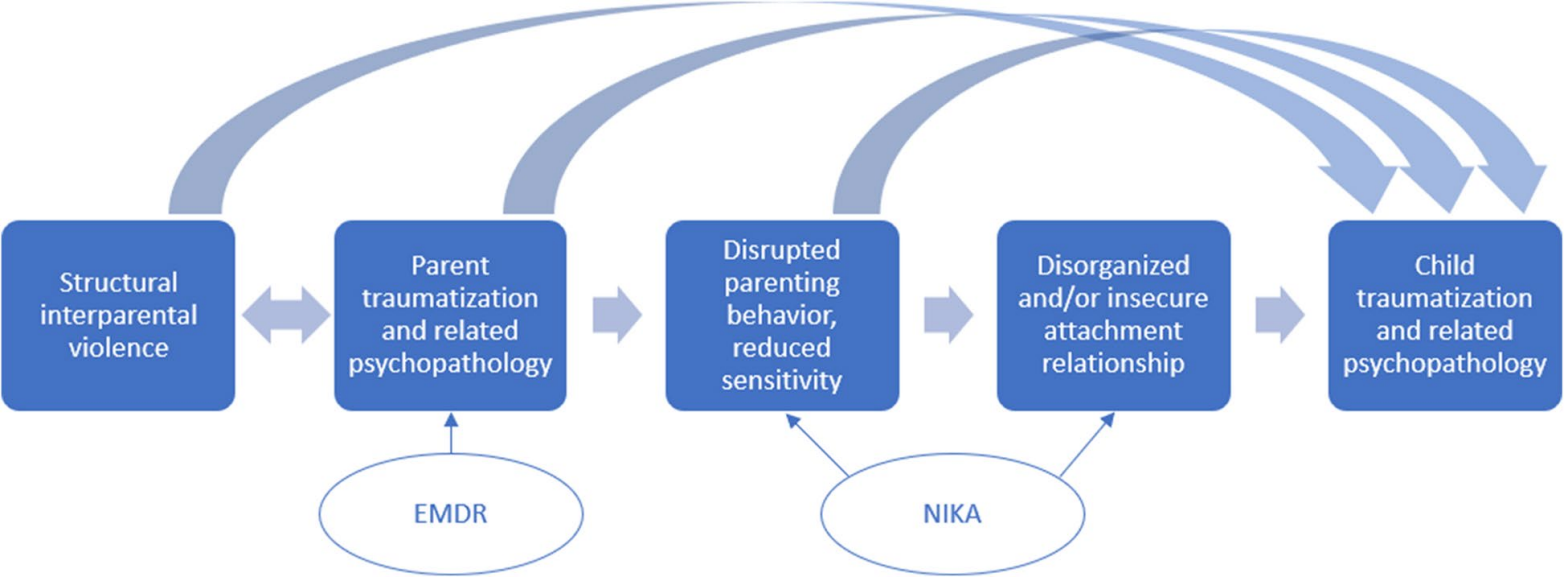
Conceptual overview of the interplay between interparental violence, parent traumatization, and parenting behavior, and how this may affect child development. The circles indicate the interventions that are tested in this study

In order to reduce the impact of interparental violence on young children, the parent-child relationship might be a key target for intervention [15, 16]. For victimized parents who have been abused by their (ex-)partner, parenting may become extremely challenging due to post-traumatic stress disorder (PTSD) symptoms and related psychopathology [10, 17]. Children who are exposed to interparental violence and related adversities have a high risk of developing psychopathology as well, including PTSD [18, 19]. A secure attachment relationship can protect children from developing maladaptive psychological outcomes [20–22], also after experiences of child maltreatment [23, 24]. While children who have been exposed to interparental violence would therefore urgently need a stable and nurturing attachment relationship as a buffer to protect them from (further) developing trauma-related symptomatology, in reality, such caregiving is in short supply. Victimized parents who suffer from PTSD symptoms and related psychopathology are often unable to support their child and they are more likely to show frightened and frightening behaviors towards their child, which compromises treatment and healthy development of the child [25, 26]. An attachment-based parenting program would therefore be vital to promote secure attachment interactions between traumatized parents and their children, which would consequently result in a reduction of children’s PTSD symptoms and related psychopathology [27–29]. See Fig. 1 for a conceptual overview.

To this moment, very few randomized controlled trials (RCTs) have been conducted specifically aimed at preschool children who have been exposed to interparental violence. One such study is a trial that investigated the effect of Child-Parent Psychotherapy (CPP) in a sample of mothers and children between the age of 3 and 5 years old who had witnessed interparental violence [16, 30]. CPP is an attachment-based intervention that is conducted through weekly sessions for the duration of 1 year. This intervention adopts a trauma-informed approach: the development of a joint trauma narrative of the parent and child is a central element throughout the intervention. The RCT showed that CPP led to a reduction of trauma-related symptomatology in both children and their mothers. Unfortunately, in this study, the quality of parent-child interactions was not assessed. This implies that we can only speculate that the effects on child and parental trauma symptoms occurred through an improved parent-child relationship, since this is a main focus of the intervention program. Nevertheless, this trial showed that CPP is a suitable intervention for traumatized mothers with young children. A disadvantage of this intervention is that it is quite lengthy (weekly sessions for the duration of 1 year). Besides the high costs and long duration of this intervention, CPP demands that certain conditions are met, such as a safe and stable family situation over a relatively long period of time, which limits the implementation of this program in practice. Several studies evaluating attachment-based interventions for parents (at risk for) child maltreatment have shown that short-term attachment-based interventions (duration shorter than 3 months) can also be effective in promoting the quality of parent-child interactions and children’s well-being [31–33]—although such optimistic findings are not consistently reported [34, 35]. Advantages of short-term attachment-based interventions are that the costs are relatively low and that completing these interventions in this hard-to-reach population of families is less challenging. Another drawback of the CPP trial is that children younger than 3 years old were not included, even though CPP was designed for younger children as well. It has thus not yet been empirically investigated how the needs of traumatized infants and toddlers who have been exposed to interparental violence can be best addressed.

In the current study, we will advance the current knowledge on effective treatment of this population by evaluating the effectiveness of NIKA, a video-feedback intervention aimed at enhancing the attachment relationship, in a sample of victimized parents and their young children (0.5–6 years old) after severe interparental violence. This intervention was selected for this study in a preliminary study that was conducted prior to the trial [36]. This preliminary study involved a literature search and an expert meeting involving a group of therapists with ample experience in conducting trauma- and attachment-based interventions in the Netherlands. The main practical reasons to select NIKA for this study were that this intervention was developed specifically for families after interparental violence or other types of child maltreatment and that NIKA is already being used in several domestic violence shelters throughout the Netherlands, which will likely facilitate implementation of the current study’s results in Dutch practice.

#### The importance of addressing parental PTSD symptoms

Adults who are victims of interparental violence are at an increased risk for developing PTSD [37, 38]. This risk is further exacerbated by the fact that many victimized adults have a history of chronic and interpersonal traumatization themselves, for instance resulting from experiences of childhood maltreatment [39, 40]. In line with the concept of relational PTSD, it might be expected that if parents show reduced PTSD symptoms, for example after receiving trauma therapy, this would lead to improved parent-child interactions, more security for the child, and therefore a reduction of PTSD symptoms in the child as well. Following this line of reasoning, it would be important that parents who suffer from PTSD symptoms and related psychopathology receive trauma therapy to reduce their own PTSD symptoms—among which those symptoms that interfere with the parent-child relationship.

For individual treatment of adult PTSD, meta-analytic evidence consistently demonstrates the effectiveness of EMDR therapy and trauma-focused cognitive behavior therapy (TF-CBT) [41–45], also for adults with PTSD resulting from childhood maltreatment [46]. Both interventions are therefore recommended in several Dutch and international guidelines [47–49]. Nevertheless, several arguments favor the use of EMDR therapy relative to TF-CBT in the specific context of this study. First, a recent economic analysis showed that EMDR therapy is the most cost-effective trauma intervention for adults [50]. In addition, in the expert meeting that was organized for our preliminary study, the large majority of experts considered EMDR therapy as the most appropriate therapy for victimized parents who have experienced interparental violence, because this is an efficient therapy that leads to PTSD symptom reductions in a short time frame [36]. To our knowledge, the effectiveness of EMDR therapy (or any other trauma therapy) for the specific group of victimized parents after interparental violence has not yet been empirically investigated. In the current study, we aim to fill this gap by evaluating whether EMDR therapy can be used to reduce PTSD symptoms in this population.

#### Synergetic effects of attachment-based intervention and individual trauma therapy for the victimized parent

Because of the complementary primary foci of NIKA and EMDR therapy, it would be valuable to explore the synergetic effects of these two interventions. While NIKA adopts a trauma-sensitive approach by explicitly linking parents’ past traumatic experiences to dysfunctions in their current parenting behavior, for parents who suffer from PTSD symptoms, their ongoing trauma symptomatology might still be an interfering factor in the relationship with their child. Individual trauma therapy focused on reducing their own PTSD symptoms might then be additionally needed in order to further improve the parent-child relationship. Hence, we hypothesize that if parents with clinical PTSD symptoms additionally receive EMDR therapy after NIKA, they would show an even greater decrease in disrupted parenting behavior and a greater increase in sensitive parenting behavior compared to parents with clinical PTSD symptoms who only receive NIKA. Considering the direct and indirect impact of parental PTSD symptoms and related psychopathology on the development of young children, this should also be reflected in a stronger decrease in PTSD symptoms and related psychopathology of the child. This assumption has not yet been empirically tested, but several studies did investigate whether parental traumatization hampers the effectiveness of attachment-based parenting interventions. Although one study did not confirm this hypothesis [51], three other studies did show that traumatized parents responded less well to these interventions [52–54]. These latter findings support the assumption that individual trauma therapy focused on the parent, in addition to an attachment-based intervention such as NIKA, would generate increased effects.

To our knowledge, there is currently no specific evidence available concerning the most effective order in which to offer attachment-based treatment versus parent-focused trauma therapy (if there even is one specific order which would work best for all families). Therefore, we relied on clinical experience to determine the order of these two interventions in this study. We chose to start by offering NIKA, followed by EMDR, for several reasons. First, parents who receive treatment from a domestic violence shelter are often dismissive towards their own psychological distress (so-called survival mode) and therefore less open to receive treatment focused on reducing their own PTSD symptoms. They are usually more motivated to receive treatment that involves their child(ren), as is the case with an attachment-based intervention such as NIKA. Throughout the NIKA sessions, parents become increasingly aware of the impact of their own (potentially disrupted) behaviors on their child and the link between these behaviors and their own traumatic experiences. In addition, after NIKA the child’s behavior likely becomes easier to handle, which might leave the parent with more mental space to engage in a trauma therapy such as EMDR. Hence, we expected that, even though this order might not be the best order for every dyad, this order would be the most generic. In a separate study, we aim to generate more knowledge on determining the best order of attachment- and trauma-based treatment components for this population.

### Objectives {7}

The goal of this study is to examine the effectiveness of a video-feedback intervention aimed at enhancing the parent-child relationship (NIKA), individual trauma therapy for parents (EMDR therapy), and the combination of both interventions in parent-child dyads who have experienced severe interparental violence. Our primary hypotheses are (1) parents who receive NIKA will show a stronger increase in disrupted parenting and a stronger increase in sensitive parenting than parents in the waitlist usual care group and (2) parents with clinical PTSD symptoms who receive EMDR therapy will show a stronger decrease in PTSD symptoms than parents in the waitlist usual care group. In addition, a secondary hypothesis is that children whose parents receive NIKA will show a stronger decrease in PTSD symptoms and emotional and behavioral problems than children in the waitlist usual care group. Finally, our secondary hypotheses concerning the synergetic effects of NIKA and EMDR therapy are that parents with PTSD symptoms who receive NIKA combined with EMDR therapy will show (1) a stronger decrease in disrupted parenting and (2) a stronger increase in sensitive parenting than parents who only receive NIKA and (3) that their children show a stronger decrease in PTSD symptoms and comorbid symptomatology than children whose parents only receive NIKA.

### Trial design {8}

**Fig. 2:**
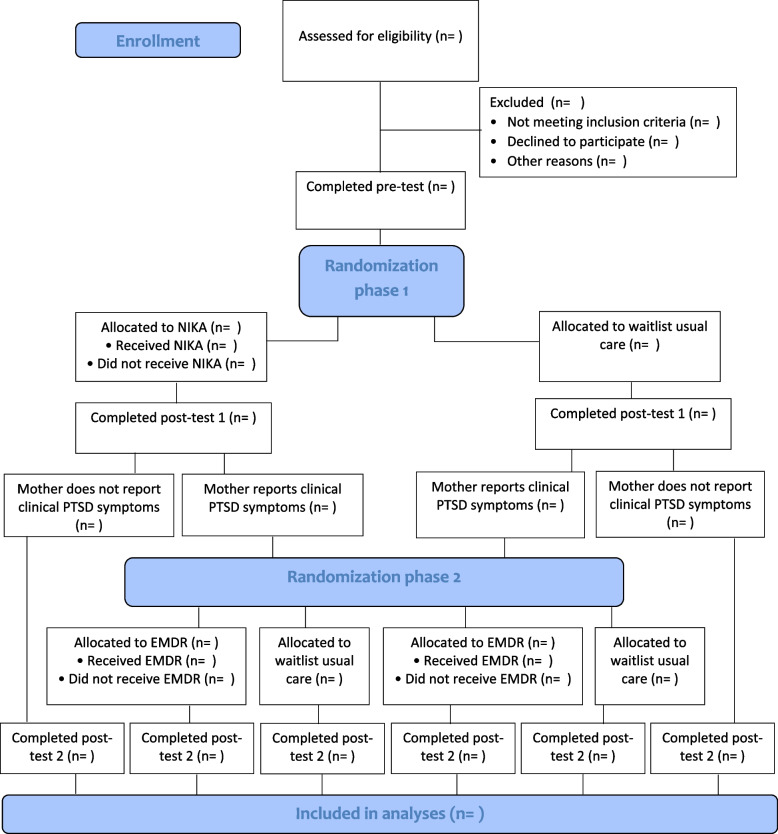
CONSORT flow diagram

A multicenter randomized controlled superiority trial with parallel groups is conducted. Figure 2 provides an overview of the study design. The trial consists of two phases. During the first phase, eligible dyads are randomly assigned to either NIKA or a waitlist usual care group. A pre-test is conducted prior to the treatment period and a post-test takes place directly afterwards (6 weeks after the pre-test). Directly after the post-test of phase 1, parents who report clinical PTSD symptoms are randomly assigned to either EMDR therapy or a waitlist usual care group. Six weeks later, a post-test of phase 2 is conducted for all participating dyads. The total study duration for each participating dyad is 13 weeks.

## Methods: participants, interventions, and outcomes

### Study setting {9}

The trial is conducted in multiple domestic violence shelters in the Netherlands. All research appointments and treatment sessions are carried out in the domestic violence shelter or, in case treatment is continued through ambulatory care, at the dyad’s home. The therapists and researchers travel to these locations.

### Eligibility criteria {10}

In order to be eligible to participate in this study, a dyad must meet all of the following criteria:

- The parent and child are residing in a domestic violence shelter because of interparental violence between the residing and non-residing parent (because the residing parent is usually the mother and the non-residing parent usually the father, for readability purposes, we will refer to the residing parent as the mother and to the non-residing parent as the father in this protocol)
- The child is aged between 6 months and 6 years old (if there is more than one child in this age range in the family, the youngest child between 1.5 and 6 years old will be selected to participate in the study)
- The mother speaks Dutch or English sufficiently to be engaged in the treatment program and reliably respond to the questionnaires, or a professional interpreter is available. Enrolled in

Potential dyads are excluded from participation in this study if the mother has a serious mental illness other than PTSD (e.g., psychosis) that requires immediate intervention.

### Who will take informed consent? {26a}

If a dyad that is eligible for participation in this study is residing in one of the participating domestic violence shelters, the mother is first informed about the study by the involved social worker. The social worker provides the mother with a short information flyer about the study. If the mother is interested in participating in the study, one of the researchers travels to the domestic violence shelter to provide an oral explanation of the study and to give the mother a detailed information letter about the study. The mother has a couple of days to consider her decision about participating in the study. If the mother agrees to participate, the mother is asked to sign for informed consent. In case the father has custody over the child as well, we follow a strategy that is in line with the current practice in the domestic violence shelters by asking the mother to contact the father. If the mother does not want to contact the father herself, but does allow the researcher to contact the father, one of the researchers contacts the father. In case the father does not respond within 2 weeks (despite multiple efforts of the researchers and/or mother to contact the father), the mother is asked whether the father has let her know in any other way that he would not approve of the child’s participation. If this is the case, the mother and child are excluded from the study. If this is not the case, the mother and child can participate in the study. This approach for allowing children to participate in a study with permission from only one of the two custodial parents when the other custodial parent is unreachable has been approved by the medical research ethics committee, similar to a previous study that was conducted in a similar setting in the Netherlands [55].

### Additional consent provisions for collection and use of participant data and biological specimens {26b}

At the informed consent form, parents can indicate whether or not they give their permission to use their data for future studies and whether or not they give their permission to be contacted by the researchers for future studies. No biological specimens are collected in this trial.

## Interventions

### Explanation for the choice of comparators {6b}

All dyads receive care as usual (CAU) in the domestic violence shelter. The comparison group in this study is placed on a waitlist for NIKA and, if applicable, EMDR. The main reason for this choice is that it would be unethical to withhold the participating dyads completely from these interventions. In addition, the domestic violence shelter already offers an environment in which families receive support to deal with the consequences of domestic violence. Mothers and their children usually reside in the domestic violence shelter for a period of 6 weeks to 9 months, depending on the domestic violence shelter organization and the possibilities for transitioning to permanent housing. After this period, further treatment is continued through ambulatory care. The goal of the guidance that is provided to women in the domestic violence shelter is to develop a safe environment and to learn how to become independent and how to prevent becoming involved in interparental violence again in the future. The standard guidance that is provided in the different domestic violence shelters is roughly similar: a social worker is involved with the family throughout their stay in the domestic violence shelter. This social worker also involves the social network, and, if possible, the non-residing parent in the treatment plan for the family. In addition, help is provided to mothers with respect to practical issues such as financial problems, schooling for the child and mother, legal issues, and future housing of the family. In addition to individual support, group activities (e.g., support groups for mothers) are regularly organized. Even though the participating domestic violence shelter organizations have slightly different emphases, both organizations use nationally available protocols for support in domestic violence shelters [56, 57]. Currently, these protocols do not include an assessment of the need to provide trauma- and attachment-based interventions nor routine offering of such intervention. If the mothers or children need more specialized support, this is provided by either an external youth care organization or by psychologists from the domestic violence shelter.

### Intervention description {11a}

#### NIKA

NIKA is a short-term video-feedback intervention aimed at enhancing the parent-child relationship for parents and their children aged between 0 and 6 years old [14]. The intervention specifically targets high-risk families, for instance, families where child maltreatment or other forms of domestic violence have occurred. The goal of NIKA is to prevent or reduce disorganized attachment and related psychopathology in children by reducing disrupted parenting behavior and increasing sensitive parenting. The intervention uses elements from cognitive behavior therapy and is based on attachment theory. Even though NIKA has similar elements to several existing attachment-based video-feedback interventions (e.g., Basic Trust [58] and Video-feedback Intervention to promote Positive Parenting and Sensitive Disciplining, VIPP-SD [59]), NIKA has a stronger emphasis on diminishing disrupted patterns in the attachment relationship. NIKA was developed by ND and KZ and is already in use in several domestic violence shelters in the Netherlands. The NIKA manual includes the theoretical background of the intervention and a clear protocol to support therapists during the intervention. It also includes several working materials for parents (e.g., a homework assignment) to help them transfer their newly acquired skills to their everyday life.

NIKA is provided through a structured protocol. NIKA starts with an assessment session consisting of an attachment interview, a questionnaire, and a video observation. For the video observation, the parent and child are asked to play together for a couple of minutes while a few mild stressors are added. In this trial, the video recordings that are made during the pre-test are used for this purpose (stressors in this session include the use of age-inappropriate toys, a too large variety of toys, and the instruction to clean up the toys after a couple of minutes). The goal of the assessment is to get acquainted with the child’s attachment behaviors, the disrupted patterns that are present in the parent-child relationship, and the roots of these patterns in the parent’s own trauma history, as well as the parent’s reflective functioning and sensitive behaviors. After the assessment session, several video-feedback sessions take place. For this trial, we standardized the number of video-feedback sessions so that each dyad will receive four video-feedback sessions of 1 h each. These sessions have a similar structure: first, the therapist helps the parent formulate a personalized goal and reflect on the parent’s interactions with the child. The therapist and parent then discuss the video recordings that were made during the previous session. The therapist uses psycho-education (supported with visual materials) to explain what sensitive parenting behavior is and helps the parent reflect on his or her parenting behavior based on the psycho-education and video recordings. Throughout the session, the therapist stimulates the parent to mentalize about the child’s behaviors, thoughts, and feelings in the video recordings (e.g., “What is your child thinking right now?”). In case the parent has dysfunctional cognitions about the child (e.g., if the child is playing on its own and the parent interprets this behavior as if the child is rejecting him or her), cognitive restructuring techniques are used to change these cognitions. The parent is reinforced by the therapist whenever the parent displays adequate insights or reflections. To provide the parent with feedback on disrupted parenting behavior, the therapist selects a fragment that reflects a disrupted pattern in the parent-child relationship. The video is first paused at a moment where the child shows attachment-eliciting cues. The parent is asked what the child would feel, think, and need in this instant. Subsequently, the disrupted parenting behavior that follows in the video recording is displayed to the parent. The therapist reflects on this moment with the parent by discussing the relation between this parenting behavior and the parent’s own traumatic history. The parent is explained what the negative effect of this behavior is on his or her child, by showing the child’s response in the video recording and providing the parent with psycho-education. The therapist also gives the parent specific advice on how to improve the disrupted parenting behavior. At the end of the feedback session, the therapist shows a fragment in which the parent displays sensitive parenting behavior. This way, the parent is reinforced in his or her abilities as a parent and feels more confident. In the final session, the assessments of the intake session are repeated and the therapist and parent reflect on the effect of the feedback sessions.

#### EMDR therapy

Only parents who report clinical PTSD symptoms assessed with the PTSD Checklist for the DSM-5 (PCL-5, score ≥31 [60]) during post-test 1 (see Fig. 2) are randomized to one of the trauma therapy conditions (EMDR versus waitlist usual care). For a more detailed explanation of this instrument and the cut-off score that was used, see the “Outcomes {12}” section. EMDR therapy is a protocolized, eight-phase trauma therapy, which aims to reduce symptoms resulting from disturbing and unprocessed traumatic experiences. This method includes history taking, preparation, assessment, desensitization, installation, body scan, positive closure, and reevaluation [61]. Before the onset of the EMDR sessions, a case conceptualization is made with the parent (1 session of max 90 min) to identify the traumatic events that are currently related to their PTSD symptoms and have the most negative impact on the parent-child relationship. These may be traumatic experiences during and before parenthood, for example during childhood. Memories will be placed in a hierarchy based on the Subjective Units of Disturbance (SUD) and are treated subsequently from high to low SUD. During treatment, patients are asked to memorize the most distressing part of the experienced traumatic event, including images, thoughts, emotions, and physical sensations. At the same time, their working memory capacities are challenged by visually tracking the horizontally moving finger of the therapist (or a light bar) or other stimuli to maximize the workload of the working memory, such as hand-held buzzers. For a full description of the treatment protocol, see Shapiro [61]. In the current trial, the Dutch version of the standard EMDR protocol is used [62] and a maximum of 5 weekly sessions of EMDR therapy will be applied with a duration of 90 min each. It is assumed that some of the participants are able to process multiple memories per session. Therefore, early completers are expected in this study. Treatment completion is defined as receiving five sessions, or less if (a) SUD scores are zero for all identified traumatic memories and (b) there is an agreement between the parent and therapist that the PTSD symptoms are sufficiently reduced to warrant terminating treatment.

### Criteria for discontinuing or modifying allocated interventions {11b}

In case a mother refuses NIKA or EMDR, or does not want to complete all treatment sessions, we still aim to complete the research assessments for the dyad.

### Strategies to improve adherence to interventions {11c}

We apply several strategies to optimize treatment adherence in this trial. All NIKA therapists have a university-level degree. Training for NIKA involves a 3-day course, followed by a supervision trajectory and e-learning modules. For EMDR, trial therapists are licensed clinical psychologists who completed an EMDREA (EMDR Europe Association)-accredited EMDR training (basic and advanced level).

During this trial, all therapists will attend monthly supervision groups of 1 h for both NIKA and EMDR. One of the NIKA developers (KZ or ND) and an EMDREA-approved consultant (CdR) will review videotapes of NIKA and EMDR sessions. KZ and CdR will also review all case conceptualizations and session checklists for NIKA and EMDR, respectively. Additional supervision will be provided via email and telephone upon request. Finally, 10% of the videotapes of treatment sessions will be randomly selected, stratified on therapist and session, and rated for adherence, by trained Master students who are blinded to outcome and trained to assess adherence using NIKA- and EMDR-specific fidelity checklists.

### Relevant concomitant care permitted or prohibited during the trial {11d}

Participating dyads do not receive parent-child interventions (aside from some general forms of parenting support, such as psycho-education) or specialized trauma therapy during the 13 weeks of their participation in the study other than NIKA and EMDR therapy if they are allocated to these interventions.

### Provisions for post-trial care {30}

Not applicable, post-trial care will not be necessary for the participating dyads because they receive care from the domestic violence shelter throughout and directly after participating in this study. Dyads who were allocated to the waitlist usual care group will receive NIKA and EMDR directly after participating in this study.

### Outcomes {12}

The main parameters of this study are disrupted parenting behavior, sensitive parenting behavior, and PTSD symptoms of the mother. Disrupted parenting and sensitive parenting behavior are measured based on a 10-min mother-child observation, with the Atypical Maternal Behavior Instrument for Assessment and Classification (AMBIANCE) [63] and Ainsworth Sensitivity Scale [64], respectively. These observational instruments were chosen for this study for several reasons. First, these observational scales are both rooted in attachment theory and fit well with the goals of NIKA, during which the mothers receive explicit feedback on both disrupted and sensitive parenting behaviors. Second, by using observational instruments rather than self-reported parenting behavior, we obtain a less biased view of the quality of parenting behavior. Finally, both measures have good psychometric properties and are commonly used in our field. In the analyses, we will compare changes in parenting behavior from the pre-test to the first post-test between the two conditions (NIKA versus waitlist usual care), see the “Sample size {14}” section for more details about the analyses.

Maternal PTSD symptoms are measured with a self-report questionnaire, the PCL-5 and Life Events Checklist for the DSM-5 (LEC-5) [60]. We chose this instrument, because this is a widely accepted self-report measure of PTSD symptoms [65] and this version is consistent with the most recent version of the DSM (DSM-5). International validation studies have shown excellent psychometric properties of this questionnaire [66, 67]. Recently, a preliminary validation study also demonstrated good reliability and validity in the Netherlands [68]. Because of practical reasons (i.e., time to conduct a more elaborate measure and burden of the mothers during the research appointments), and because we are interested in the overall amount of distress mothers experience due to their previous traumatic experience, rather than whether or not they have an official diagnosis of PTSD, we decided not to use a more extensive measure of PTSD (such as the Clinician-Administered PTSD Scale for DSM-5 [69]). Because the PCL-5 had not yet been validated in a Dutch population at the time this study started, we relied on international validation studies to determine a cut-off score for clinical PTSD symptoms [66, 67]. These studies showed that cut-off scores between 31 and 33 are most predictive of PTSD. Because the goal of our study is to reduce clinically meaningful PTSD symptoms and related psychopathology in mothers, we decided to use the lower side of this range as a cut-off for clinical PTSD symptoms (score of 31). A sum score of the PCL-5 will be used in the analyses for each participant. We will compare changes in maternal PTSD symptoms from the first post-test to the second post-test between two conditions (EMDR versus waitlist usual care), see the “Sample size {14}” section for more details about the analyses.

The secondary outcomes of this study are PTSD symptoms and emotional and behavioral problems of the child. PTSD symptoms of the child are assessed with the Child and Adolescent Trauma Screen (CATS, version for 3–6 years [70]), which is filled out by the mother and involved social worker. This questionnaire is only used for children older than 12 months. We chose this measure because this measure is, to our knowledge, the only internationally validated screening questionnaire for PTSD symptoms in preschool children that is consistent with DSM-5. In addition, the same (practical) arguments as for our rationale to choose for the PCL-5, apply for our decision to work with the CATS rather than a more extensive measure of children’s PTSD symptoms (such as the Diagnostic Infant and Preschool Assessment [71]). The CATS has been validated for samples of children aged 3–6 years in the USA, Germany, and Norway [70]. The CATS has not yet been validated in a Dutch sample (a validation study is currently ongoing), nor for children younger than 3 years. We will compare changes in child PTSD symptoms (sum score, aggregated for the two informants) from the pre-test to the first post-test between two conditions (NIKA versus waitlist usual care), see the “Sample size {14}” section for more details about the analyses.

For the assessment of emotional and behavioral problems, the Child Behavior Checklist (CBCL) 1.5–5 years [72] is filled out by the mother and the Teacher Report Form (TRF) 1.5–5 years [72] is filled out by the involved social worker or another professional who is involved with the child, such as a caregiver from a child care center. Similar to the CATS, these questionnaires are only used for children older than 12 months. We chose these Achenbach System of Empirically Based Assessment (ASEBA) forms because they are widely used throughout the world and have well-documented psychometric properties. We will compare changes in emotional and behavioral problems (sum score, aggregated for the two informants) from the pre-test to the first post-test between two conditions (NIKA versus waitlist usual care), see the “Sample size {14}” section for more details about the analyses.

Furthermore, several background variables are assessed in this study: demographic variables (e.g., age, gender, SES), parental psychopathology (assessed at all time points with the Brief Symptom Inventory (BSI)-18 [73]), the maltreatment history of the child (assessed with the Modified Maltreatment Classification System [MMCS] based on case file coding [74]), parental childhood trauma (assessed during pre-test with the Childhood Trauma Questionnaire Short Form; CTQ-SF; [75]), and received professional support (e.g., other types of therapy provided to the family during the study, assessed based on case file coding). In addition, we will document all relevant characteristics of the participating domestic violence shelters (e.g., type of care that is provided to the families) and therapists (e.g., work experience, treatment fidelity).

### Participant timeline {13}

**Fig. 3:**
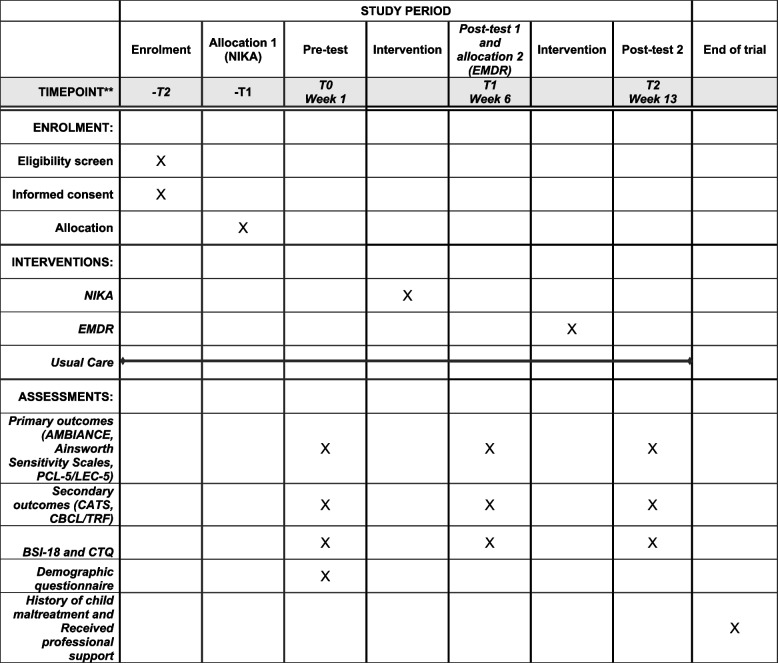
Schedule of enrolment, interventions, and assessments

The total study duration for each participating dyad is 13 weeks. After informed consent is obtained, the mother and child are asked to participate in the pre-test, during which the mother is asked to fill out several questionnaires and 10 min of parent-child interactions are videotaped. In addition, we ask a social worker in the domestic violence shelter to fill out a questionnaire about the child. Prior to the pre-test, the dyad is allocated to NIKA or the waitlist usual care group (the dyad will be notified about this directly after the pre-test). NIKA consists of one intake and four 1-h sessions, divided over 5 weeks. During this time, dyads in the waitlist group only receive CAU in the domestic violence shelter. In week 7, a post-test is conducted, which is similar to the pre-test. Phase 2 of the design starts in that same week. Mothers who report clinical PTSD symptoms at the post-test are allocated to EMDR or the waitlist usual care group. EMDR consists of one intake and a maximum of five 90-min sessions over the course of 5 weeks. Mothers in the waitlist group only receive CAU during this period. Mothers who do not report clinical PTSD symptoms in phase 2 are not included in the randomization. They will still be involved in the post-test of phase 2, to prevent dropout bias (i.e., by making sure the investment for this study will be the same for each participating dyad, it is not likely that dropout rates will differ for the conditions). In week 13, the post-test of phase 2 takes place for all dyads, which is similar to the previous assessments. All assessments take place in the domestic violence shelter or, in case the dyad’s treatment is continued through ambulatory care, at the dyad’s home. The assessments are conducted by two trained (assistant) researchers and take approximately 1 h each. See Fig. 3 for an overview of the timeline for each dyad.

### Sample size {14}

The planned sample size for this study is *N* = 150. Separate power calculations for the two study phases have been conducted.

#### Phase 1

We conducted a power analysis for longitudinal linear mixed effect models with unbalanced designs in R using the “powerlmm” package [76]. Based on recent meta-analyses regarding the effectiveness of trauma-informed parenting interventions after violence exposure [77] and a short-term attachment-based intervention that includes some components similar to NIKA [78], we assumed an effect size of *d* = 0.50 for the effects of NIKA on disrupted and sensitive parenting. We estimated three-level models accounting for repeated measures over time (level 1) and partial nesting of families (level 2) within therapists (level 3) (only in the NIKA group). We estimated approximately 50% variance to occur between subjects and 5% variance between therapists. For a sample of *N* = 150 (*n* = 75 per group), two time points, and two-sided testing with a significance level of *α* = .05, we will have 86% power to detect effects of *d* = 0.50.

#### Phase 2

The estimated effect size of EMDR therapy on PTSD symptoms of the mother is based on a recent meta-analysis in which effect sizes of EMDR therapy were reported within a range of *g* = 0.67–1.18 [79]. Because the current implementation of EMDR therapy (focus on memories of traumatic events that are related to their PTSD symptoms and also hinder positive parent-child interactions) is different from previous studies, we decided to stay on the conservative side of this range by assuming an effect size of *d* = 0.60 for this study. We estimated three-level models accounting for repeated measures over time (level 1) and nesting of mothers (level 2) within the previous condition (NIKA versus waitlist) (level 3). We estimated approximately 50% variance to occur between subjects and 5% variance between previous conditions (NIKA versus waitlist). For a sample of *N* = 80, (*n* = 40 per group), two time points, and two-sided testing with a significance level of *α* = .05, we will have 75% power to detect effects of *d* = 0.60. With a sample of *N* = 150 in phase 1, this would imply that 53% of the mothers who are recruited for this study should report clinical trauma symptoms in order to be included in the randomization for phase 2. We made this estimation based on a recent report which revealed that all women in one of the participating domestic violence shelter locations experienced severe and chronic domestic violence and 75% of the women report traumas of their own childhood [80].

To explore the synergetic effect of NIKA and EMDR therapy, a two-level analysis (only accounting for repeated measures over time, thus not considering nesting of families within therapists), estimating 80% variance to occur between subjects, we will have 68% power to detect effects of *d* = 0.50.

### Recruitment {15}

In each participating domestic violence shelter, a social worker or therapist who coordinates the flow of incoming and outgoing clients in the shelter is appointed as the contact person for this study. The researcher receives a frequent (weekly or bi-weekly) update of potentially eligible new dyads (mothers with a child aged 0.5–6 years old). In order to perform a more thorough eligibility screening of the dyad, the researcher then contacts the involved social worker for this dyad. This way, we make sure that each dyad with a child in the age range of this study is screened for eligibility for this study and each eligible dyad has the chance to participate. Recruitment of dyads started in December 2020 and will take place until July 2023.

## Assignment of interventions: allocation

### Sequence generation {16a}

In both phases of the RCT, dyads are randomly assigned to one of the two conditions. Randomization occurs with a random number generator in Microsoft Excel (1:1 allocation), stratified by the participating domestic violence shelter locations and using permuted blocks. The first randomization (NIKA versus waitlist CAU) is done prior to the pre-test by a researcher who will not conduct the pre-test. The second randomization (EMDR therapy versus waitlist CAU) is done after post-test 1. Only mothers who report clinical PTSD symptoms on the PCL-5 (for a more detailed description of this questionnaire and the cut-off score that is used, see the “Outcomes {12}” section) are randomized, according to a similar procedure in Microsoft Excel.

### Concealment mechanism {16b}

The first randomization (NIKA versus waitlist CAU) is concealed from the (assistant) researcher who conducts the pre-test, the mother, and the therapist through a closed envelope. For the second randomization (EMDR versus waitlist CAU), concealment is not necessary because the randomization is only performed after the post-test depending on mothers’ self-reported PTSD symptoms.

### Implementation {16c}

The first randomization (NIKA versus waitlist CAU) is performed prior to the pre-test by a researcher who will not conduct the pre-test with the dyad. The (assistant) researcher who conducts the pre-test is unaware of the condition the dyad is in and brings a closed envelope to the pre-test. This (assistant) researcher opens the envelope with the mother at the end of the pre-test and then informs the involved therapist. The second randomization (EMDR versus waitlist CAU) occurs after the first post-test by one of the researchers. The mother and therapist are informed about the condition once the researcher has performed this randomization sequence as described above.

## Assignment of interventions: blinding

### Who will be blinded {17a}

Participating mothers and their therapists and social workers cannot be blinded for study condition in this trial, because the control group is placed on a waitlist. The research assessments only include self-report questionnaires (in the presence of a researcher) and a video observation with no interference from the researcher. Therefore, it is not necessary to blind the researchers who perform the assessments to the study condition of the dyad. All observational data in this study will be coded by researchers who are blind to the condition of the dyads.

### Procedure for unblinding if needed {17b}

A procedure for unblinding is not needed in this study, because the participants and involved social workers and therapists are aware of the condition the dyad is in.

## Data collection and management

### Plans for assessment and collection of outcomes {18a}

At all three time points, the mother-child dyad is invited for a 1-h research appointment. All research appointments are similar in structure and content. After a short explanation of the (assistant-)researcher, the mother is asked to fill out a general questionnaire (only during the pre-test), the BSI [73], and CATS [70]. Then, a 10-min free play observation is conducted and videotaped. During the first 5 min of this observation, the mother and child are instructed to play together without toys as they would normally do. Next, a set of toys is provided, and the mother and child are instructed to play with these toys as they would normally do. The set of toys consists of a too large variety of toys, including a hand puppet, a toy that can make much noise, and age-inappropriate toys. Also, ambiguous toys (such as a spider and a police car) are included. After 4 min, the mother is instructed to clean up the toys with her child. These elements were included to make the play session a bit more challenging and stressful for the mothers. After the observation task, the mother is asked to fill out the CTQ [75], LEC-5 [60], PCL-5 [60], and CBCL [72]. All questionnaires are filled out on a laptop using the survey platform Qualtrics, or, in case this has the mother’s preference, on paper. The researcher remains in the same room during the administration of the questionnaires, so that the mother can ask questions. Because some questionnaires may elicit negative emotions in the mothers, the researcher remains vigilant of any signs of distress in the mother while she is filling out the questionnaires. If this occurs, the researcher will comfort the mother and pause the research appointment as long as necessary. At the end of the research appointment, the researcher will point the mother towards the possibility to contact her social worker if she would like to talk about anything related to the questionnaires. Even though the child is present during the observation talk, whenever possible the child will not be in the same room while the mother fills out the questionnaires. In most domestic violence shelters, there is an internal child care facility where the child can stay during this time. If this is not an option, the second researcher who is present will watch the child in another room while the mother fills out the questionnaires.

In case the mother does not (sufficiently) speaks Dutch or English, a professional interpreter will be called and put on a speaker to guide the mother through the research appointment. Furthermore, all research information has been translated into English and Arabic for this study, because we expected these two languages would be the most prevalent among non-Dutch-speaking mothers. Whenever possible, validated international versions of the questionnaires are used in the language of the mother. For the CTQ [75], CATS [70], and BSI [73], no validated Arabic version was available. We translated these questionnaires for this study through a back-and-forth translation method by a native Arabic speaker and a professional Arabic translator. In case the mother has a different native language than Dutch, English, or Arabic, or the mother is analphabetic, the interpreter will provide live translations of the questionnaires to the mother during the appointment.

*Disrupted parenting behavior* is assessed at all time points based on the 10-min observation of mother-child interactions. We will use the AMBIANCE [63] to code disrupted parenting behavior. Coders will be trained through the standard AMBIANCE training and should achieve a satisfactory intraclass correlation coefficient (ICC) for the overall level of disrupted communication on a subset of videotapes from both observational tasks in this study (ICC ≥.70). In addition, regular supervision sessions will be organized throughout the coding process to prevent coder drift.

*Parental sensitivity* is assessed at all time points using the same observations as described above. We will use the Ainsworth scale for sensitivity [64]. Coders will be trained by an experienced researcher who has been previously trained in the Ainsworth scale. Similar to the AMBIANCE, coders should achieve satisfactory intraclass correlation coefficient (ICC) rates for the sensitivity scale on a subset of videotapes of this study (ICC ≥.70) and regular supervision sessions will be organized throughout the coding process.

All questionnaires that are used in this study are well-validated instruments, see the “Outcomes {12}” section for more details.

### Plans to promote participant retention and complete follow-up {18b}

We stimulate the participating dyads to complete all study assessments in several ways. We provide the mothers with a small compensation of €20, after each assessment, and the child with a small gift (age-appropriate toys). After the final research appointment, we send the mothers a compilation of the videotaped mother-child interactions through a secured link. Throughout each dyad’s research trajectory, we aim to be as flexible as possible to fit the research appointments in their schedule. If not all parts of the research appointment can be completed in one appointment, we schedule another appointment as soon as possible to finish all remaining assessments. If the dyad moves away from the domestic violence shelter during the study, we will visit the dyad in their new home for the upcoming research appointments. In the analyses, we will account for potential missing data by applying multilevel multiple imputation.

### Data management {19}

All questionnaires are collected digitally through Qualtrics. Questionnaires that are filled out on paper by the mother are entered into Qualtrics by a research assistant. During data collection, Qualtrics data are copied to an SPSS file on a weekly basis (each new version of the SPSS file is saved under a new name, including the current date). Digital data (including questionnaires, videotapes, databases, and processed data) are stored in a secured folder within the university network that is only accessible to research members and has automated back-ups. Questionnaires and videotapes that have to be shared with the NIKA or EMDR therapists for the treatment sessions never contain the names of the participants and are shared through a secured link (SURFdrive). The dyads each get an identification code, which is not based on their initials or birth date, and a subject identification list is used to link these numbers to the participants. Video recordings of therapy sessions and all therapy documents (e.g., intake and session forms) are transported to the research team by the therapists through a secured link. These data will be stored in a secured folder that can only be accessed by the researchers. The handling of the personal data complies with the EU General Data Protection Regulation and the Dutch Act on Implementation of the General Data Protection Regulation (Uitvoeringswet AVG, UAVG). Informed consent by the parent(s) is obtained to save the research data for future research. With the consent of the parent(s), all data will be stored for 10 years. All data handling and storage procedures are also extensively described in a data management plan in DMPonline.

### Confidentiality {27}

All research data are pseudonymized and stored in closed facilities within Leiden University as described under the “Data management {19}” section and are only accessible by members of the research team. The conversion key and all participant information (e.g., contact details) are stored separately from the research data in an encrypted file.

### Plans for collection, laboratory evaluation, and storage of biological specimens for genetic or molecular analysis in this trial/future use {33}

Not applicable, we do not collect biological specimens.

## Statistical methods

### Statistical methods for primary and secondary outcomes {20a}

We will conduct the analyses for this study through three-level linear mixed effect models in R (nlme, lmr, and mitml packages) that account for repeated measures over time (level 1) and nesting of dyads (level 2) within therapists (level 3). More details on the analyses are described earlier in this protocol under the “Outcomes {12}” and “Sample size {14}” sections.

### Interim analyses {21b}

No interim analyses will be conducted during this study.

### Methods for additional analyses (e.g., subgroup analyses) {20b}

To explore the synergetic effect of NIKA and EMDR therapy, a two-level analysis (only accounting for repeated measures over time, thus not considering nesting of families within therapists) will be conducted to compare dyads who have received both NIKA and EMDR, to dyads who have only received NIKA. We will compare changes in disrupted parenting behavior, sensitive parenting behavior, child PTSD symptoms, and behavioral and emotional problems from the pre-test to the second post-test between these two groups, see the “Sample size {14}” section for more details about the analysis.

### Methods in analysis to handle protocol non-adherence and any statistical methods to handle missing data {20c}

We will conduct multilevel multiple imputation [81, 82] to be able to include all dyads, including families who dropped out throughout the project, in the analyses (intention to treat).

### Plans to give access to the full protocol, participant-level data, and statistical code {31c}

We will archive our syntaxes and data documentation in Dataverse, in accordance with the guidelines for the archiving of academic research for faculties of Behavioural and Social Sciences in the Netherlands. Because the participant-level data include special categories of personal data, these data cannot be made openly accessible. However, we will share parts of the processed data that contain no personal information upon request.

## Oversight and monitoring

### Composition of the coordinating center and trial steering committee {5d}

This multicenter trial is coordinated by the researchers from Leiden University. LRAA is the principal investigator of this study. ARK coordinates recruitment and data collection and remains in close contact with the contact person of each participating domestic violence shelter (at least once per week) throughout the study. A small group of core researchers, based at Leiden University, meet on a weekly basis to monitor the trial. The full research team meets on a monthly basis for this purpose. The main funder of this trial (ZonMw) serves as the steering committee and monitors the project through yearly evaluation meetings and reports.

### Composition of the data monitoring committee, its role, and reporting structure {21a}

No formal data monitoring committee was established for this study. A data manager and privacy officer from the Faculty of Social and Behavioural Sciences at Leiden University have approved the data management plan for this study and are regularly consulted by the researchers.

### Adverse event reporting and harms {22}

The families who participate in this study will receive the care they need in the domestic violence shelters. EMDR and similar short-term, attachment-based interventions to NIKA have been used extensively in other research (for an overview of EMDR studies, see, e.g., [83]; for comparable attachment-based interventions to NIKA, see, e.g., [31, 33]). No negative consequences have been reported in these studies, so we expect this to be the same for our study. In addition, both interventions are already extensively used in Dutch practice. The measurement instruments (questionnaires and parent-child observations) that will be used in this study have also been used in previous studies. In case mothers and/or children experience distress during the research appointments or intervention sessions, the researchers or therapists will provide the needed attention.

### Frequency and plans for auditing trial conduct {23}

No formal auditing is planned for this trial. Trial conduct is evaluated on a regular basis by the researchers. Independent auditing can take place randomly by national and international supervising authorities (such as the Dutch Health and Youth Care Inspectorate). In line with the medical research ethics committee’s regulations, the research team informs the committee about trial progress on a yearly basis.

### Plans for communicating important protocol amendments to relevant parties (e.g., trial participants, ethical committees) {25}

Important protocol modifications will only be implemented after the medical research ethics committee has approved an amendment to the research protocol.

## Dissemination plans {31a}

Results of this trial will be published in international and national peer-reviewed journals and presented on international and national conferences. Study participants will receive a separate information letter and infographic concerning the results. Dissemination to the clinical field will occur through a national expert platform concerning attachment and trauma in the context of domestic violence. In addition, we will implement this study’s results in (post-)academic education programs.

## Discussion

There is still a big gap in our current knowledge regarding effective treatment approaches for traumatized parents and their young children after interparental violence. In these families, there is a high risk of disrupted parent-child interactions and often both the victimized parent and child suffer from PTSD symptoms. How this extremely vulnerable group of families can be best supported is still unknown. The current RCT is the first to evaluate the effectiveness of a video-feedback intervention (NIKA) and individual trauma therapy (EMDR) in this specific sample. A unique feature of this study is that the additive effects of a parenting intervention and individual trauma therapy for parents are explored.

The study design has several vulnerabilities. First, the legal obligation to obtain informed consent from both parents with custody over the child, that is required by Dutch law, complicates recruitment for this study. The victimized parents (often the mother) and children who participate in this study have experienced severe and often chronic interparental violence and are in some cases still in acute danger for the perpetrator. In such cases, contacting and obtaining consent from the custodial father of the child is extremely challenging and sometimes not even possible in order to protect the mother and child’s safety. We attempt to maximize the number of participating dyads with a custodial father in several ways. First, we try to contact the father by several means (telephone, email, postal address) and provide a user-friendly link through which he can give his permission digitally. Whenever possible, the researcher makes an appointment with the father to inform him personally through a phone call, so that the father is extensively informed and has had the chance to ask questions. Second, in case of non-response, the involved medical research ethics committee has approved a procedure to include dyads in this study if the custodial father does not respond or cannot be reached within a given period of time.

Another potential threat to the validity of this study is that the instruments used to assess child-related study outcomes (PTSD symptoms and behavioral and emotional problems) have not been validated for the youngest participating children (<3 years for PTSD symptoms and <1.5 years for behavioral and emotional problems). Because the assessment of these constructs in infants is extremely challenging, we decided to exclude that age group (<12 months) from these assessments. This will limit the power to detect effects on child-related study outcomes due to a smaller sample size for these analyses. PTSD symptoms are still assessed in children between 1 and 3 years (with the CATS [70]) and these data will be used in a validation study for this age group.

A second limitation related to the measurement instruments of this study is that not all instruments are validated for the different languages of the participating dyads. The population of the participating domestic violence shelters is ethnically diverse. Parents who do not speak sufficient Dutch or English can still participate in this study if an interpreter is available. For the questionnaires that have been validated in different languages, whenever possible, we use the validated questionnaire in the parent’s native language. However, if the parent is analphabetic or if there is no validated questionnaire available in the parent’s native language, an interpreter reads the questions out loud to the parent. For the observations of parent-child interactions, we will ask a translator to provide subtitles to the video in case the parent and child do not interact in the Dutch or English language. Despite the potential threat this inclusive approach poses to the validity of the measurement instruments, this aspect promotes the external validity and thereby the value of this study for clinical practice.

In conclusion, this trial has the potential to gain important insights regarding evidence-based treatment for traumatized parents and their young children who are victims of interparental violence. If the interventions that are tested in this study are effective in improving the quality of parent-child interactions and decreasing both parents’ and children’s PTSD symptoms, this will lead to significant improvements in their quality of life. An important strength is that this study has been designed and is conducted in a multidisciplinary team and in close collaboration with the domestic violence shelter organizations. Other strengths include the multisite recruitment of participants, the use of validated instruments, and manualized treatments with independent fidelity checks. If the outcomes of this study provide insight in effective treatment options for this specific population, broad-scale implementation in domestic violence shelters throughout the Netherlands will be feasible*.*

## Trial status

This study is registered in Netherlands Trial Register (NTR): NL9179, version 1. Recruitment started in December 2019. We aim to complete recruitment in July 2023.

## Data Availability

The research members who are affiliated to Leiden University will have direct access to the data sets. Other research members will be given access to the data sets upon request.

## Abbreviations

AMBIANCE: Atypical Maternal Behavior Instrument for Assessment and Classification
ASEBA: Achenbach System of Empirically Based Assessment
BSI: Brief Symptom Inventory
CATS: Child and Adolescent Trauma Screen
CAU: Care as usual
CBCL: Child Behavior Checklist
CPP: Child-Parent Psychotherapy
CTQ-SF: Childhood Trauma Questionnaire Short Form
EMDR: Eye movement desensitization and reprocessing
EMDREA: EMDR Europe Association
ICC: Intraclass correlation coefficient
LEC-5: Life Events Checklist for the DSM-5
MMCS: Modified Maltreatment Classification System
NIKA: Nederlandse Interventie Kortdurend op Atypisch oudergedrag (Dutch, short-term intervention focused on atypical parenting behavior)
PCL-5: PTSD Checklist for the DSM-5
PTSD: Post-traumatic stress disorder
RCT: Randomized controlled trial
SUD: Subjective Units of Disturbance
SUDS: Subjective Units of Disturbance Scale
TF-CBT: Trauma-focused cognitive behavior therapy
TRF: Teacher report form
VIPP-SD: Video-feedback Intervention to promote Positive Parenting and Sensitive Disciplining
